# Content analysis of medical students’ seminars: a unique method of analyzing clinical thinking

**DOI:** 10.1186/1472-6920-13-156

**Published:** 2013-12-01

**Authors:** Yukari Takata, Gerald H Stein, Kuniyuki Endo, Akiko Arai, Shun Kohsaka, Yuka Kitano, Hitoshi Honda, Hidetaka Kitazono, Hironobu Tokunaga, Yasuharu Tokuda, Mikako Obika, Tomoko Miyoshi, Hitomi Kataoka, Hidekazu Terasawa

**Affiliations:** 1 University of Florida College of Journalism and Communications, Gainesville, Florida, USA; 2 Department of Medicine and Veterans Affairs Medical Center, University of Florida College of Medicine, Gainesville, Florida, USA; 3 Nagoya Daini Red Cross Hospital, Nagoya, Japan; 4 University of Florida College of Health and Human Performance, Gainesville, Florida, USA; 5 Keio University School of Medicine, Tokyo, Japan; 6 St Marianna University School of Medicine, Kanazawa, Japan; 7 Teine Keijinkai Hospital, Sapporo, Japan; 8 Noguchi Bayside Hospital, Chiba, Japan; 9 Department of Emergency Medicine, University of Fukui Faculty of Medical Sciences, Fukui, Japan; 10 Institute of Clinical Medicine, Graduate School of Comprehensive Human Sciences, University of Tsukuba School of Medicine, Mito, Ibaraki, Japan; 11 Center for Graduate Medical Education, Okayama University Medical School, Okayama, Japan; 12 Department of Medical Education and Primary Care, Okayama University Medical School, Okayama, Japan

**Keywords:** Problem-based learning, Communication measurement, Clinical thinking development

## Abstract

**Background:**

The study of communication skills of Asian medical students during structured Problem-based Learning (PBL) seminars represented a unique opportunity to assess their critical thinking development. This study reports the first application of the health education technology, content analysis (CA), to a Japanese web-based seminar (webinar).

**Methods:**

The authors assigned twelve randomly selected medical students from two universities and two clinical instructors to two virtual classrooms for four PBL structured tutoring sessions that were audio-video captured for CA. Both of the instructors were US-trained physicians. This analysis consisted of coding the students’ verbal comments into seven types, ranging from trivial to advanced knowledge integration comments that served as a proxy for clinical thinking.

**Results:**

The most basic level of verbal simple responses accounted for a majority (85%) of the total students’ verbal comments. Only 15% of the students’ comments represented more advanced types of critical thinking. The male students responded more than the female students; male students attending University 2 responded more than male students from University 1. The total mean students’ verbal response time for the four sessions with the male instructor was 6.9%; total mean students’ verbal response time for the four sessions with the female instructor was 19% (p < 0.05).

**Conclusions:**

This report is the first to describe the application of CA to a multi-university real time audio and video PBL medical student clinical training webinar in two Japanese medical schools. These results are preliminary, mostly limited by a small sample size (n = 12) and limited time frame (four sessions). CA technology has the potential to improve clinical thinking for medical students. This report may stimulate improvements for implementation.

## Background

Learning clinical thinking is a complex task of accumulating knowledge and experience [1-3]. For medical students entering clinical training, PBL and its variations offer an entry into this complex world [4,5]. Small group discussions centered on a patient’s narrative with history, physical examination and laboratory data compromise the data, summarized as the problem list, for the discussion to extract meaningful concepts leading to diagnosis and management, loosely defined as clinical thinking. An essential component of the case discussion is the verbal communication among the students and their instructor.

Instruments to measure small group speaking as evolved a technique called content analysis (CA). CA was developed for product marketing and heath education. Borg and Gall [6] defined CA as “a research technique for the objective, systematic, and quantitative description of the manifest content of communication”. CA includes student length of speaking, participate rates, social clues, interactions, speech content to name a few of the possible measurements [7,8]. Very few medical educational CA applications have been reported [9]. A partial use of CA was explored in a recent study of the audio analysis of the ‘morning report’ of new case admissions coding the interactions between supervisors and their residents [10].

Furthermore Asian students and by inference, Japanese medical students, are particularly unaccustomed to classroom discussion, especially when led by older faculty [11]. Their hindrances include dependency and respect for authority, cultural inhibition to be silent and lack of training to ask questions that broadly include development of problem solving skills. Yet PBL requires talking as a means to learn clinical thinking [12]. Although Japanese medical education has undergone recent structural changes, little has been reported on the outcome of these changes [13]. PBL has become increasing used in Japanese medical education [14,15]. This report is the first to use CA to document medical students’ verbal responses as part of a Japanese multi-university PBL webinar developmental project.

## Methods

Content analysis of students’ verbal responses during eight webinar PBL tutoring sessions was examined.

### Participants

The study involved two US-trained clinical instructors (one female, one male) and 12 fifth year Japanese medical students (four female, eight male). The instructors were selected for their three years of general internal medicine training in the United States and their Japanese-English bilingual skills. Six randomized medical students from each of two distant Japanese medical universities were randomly assigned to one of the two instructors’ two virtual ‘classrooms’ (http://www.webex.co.jp). The study protocol specified that each instructor led four tutoring sessions (1.5 hours each) over the course of four weeks. The topics and teaching materials were standardized and designed as a syllabus by a panel of Japanese & American medical educators to promote PBL. The instructors received minimal communication skills to enhance learning; no PBL training or feedback or standardization occurred during the four tutoring sessions. All tutoring sessions had video and audio components captured for the subsequent CA. The students’ identity was masked for this analysis. All 12 students signed a detailed informed consent form.

### Code book

A CA code book to analyze the verbal interactions during the tutoring sessions.

An underlying assumption of communication CA is that dialogues are representative of underlying cognitive processes [8].

The level of critical thinking expressed by students was coded using a modified version of Practical Inquiry Model (PIM), which characterizes phases of practical inquiry through descriptors and indicators [8]. For example, the model’s second phase, Exploration, is characterized through “inquisitive” communication, which is often indicated through suggestions for consideration and brainstorming. Exploration is then followed by integration and resolution to complete the model’s phases of practical inquiry.

The PIM was organized into the code book through a series of trial coding sessions, performed by health educator coders. The coding sessions comprised analysis of speech descriptors and indicators. After the code book was finalized, an inter-coder reliability test of 19 responses, or about 10%, of all comments, involving only the variables open to interpretation, resulted in a Krippendorff’s alpha of 0.80, above the 0.75 considered acceptable [16]. Each students’ response for one of seven possible types, which essentially scaled the responses’ level of critical thinking, was coded independently by the health educator coders (Table 1). The respondent’s identity, audience, and position (i.e. whether they were the inquisitor or respondent) were also coded. The coding authors made the type critique from watching the captured recordings of the webinars; they coded all responses and comments that the students made. They made no distinction between responses and comments.

**Table 1:** Critical thinking response types

**Response types**	**Details of response types**
T1	Simple phrase responses, based on rote memory
T2	Response has more depth that Type 1 but still based on rote memory, includes *because, if, when, etc.*
T3	Response to “How would you ask a patient_______?”
T4	Response shows integration – combines ideas to form new meanings (analytical)
T5	Response shows advanced integration – combines knowledge from multiple and/or obscure sources to introduce original ideal solutions (potentially novel to instructor as well)
T6	Spontaneous questions directed to instructor
T7	Social commentary, greetings, appreciation, etc., unrelated to tutored topic

### Statistical analyses

Proportions and percentages between groups were compared using by chi-square test. Two-tailed p values less than 0.05 were considered as statistical significance. All statistical analysis was performed using SPSS-J version 20 (Tokyo, Japan).

## Results

All students’ comments, except for those related to technical issues and social commentary at the beginning and end of each session, were included in the analysis. Descriptive statistics were primarily used to characterize interactions observed in this study.

The total mean students’ verbal response time for the four sessions with the male instructor was 6.9%; total mean students’ verbal response time for the four sessions with the female instructor was 19% (Table 2). The total mean student response time for the four sessions with the male instructor was significantly lower than that with female instructor (p < 0.05).

**Table 2:** Total students’ minutes response times

	**Male instructor**	**Female instructor**	**p value**
Session 1	5.3/91 (5.8)	13.4/94 (14.1%)	0.14
Session 2	8.2/93 (8.8)	20.0/80 (25)	0.02
Session 3	4.5/78 (5.6)	17.3/90 (19.2)	0.06
Session 4	6.6/91 (7.2)	18.2/102 (17.8)	0.11

Total students’ minutes response times divided by total session time in minutes with the percent students response times in parenthesis. The total mean student response time for the 4 sessions with the male instructor was significantly lower than that with female instructor (p < 0.05).

Total students’ comments with both instructors were 458 comments. Of the total comments, responses involving the most basic level of critical thinking (T1) accounted for over half of the comments (66%, n = 302). The second level of critical thinking (T2) also accounted for the second most common response type at 15% (n = 69). Notably, the more advanced levels of critical thinking (T4 and T5), which the PBL project was striving to achieve, represented just 4% of all comments (n = 9) with no T5′s being represented.

Because the numbers of advanced levels of critical thinking responses were small, we combined the type of responses as follows: ‘simple responses’ type 1, 2 and 7; ‘advanced thinking responses’ types 3 to 5, and spontaneous topic-related questions directed to the instructor type 6 –two non topic related questions were deleted (Table 3).

**Table 3:** Combined critical thinking response types

**Response types**	**Details of response types**
Simple- Types 1, 2 and 7	Simple phrase responses, based on rote memory
	Response has more depth that Type 1 but still based on rote memory, includes *because, if, when, etc.*
	Social commentary, greetings, appreciation, etc., unrelated to tutored topic
Advanced thinking Types 3-5	Response to “How would you ask a patient_______?”
	Response shows integration – combines ideas to form new meanings (analytical)
	Response shows advanced integration – combines knowledge from multiple and/or obscure sources to introduce original ideal solutions (potentially novel to instructor as well)
Type 6	Spontaneous topic-related questions directed to instructor

**Figure 1:**
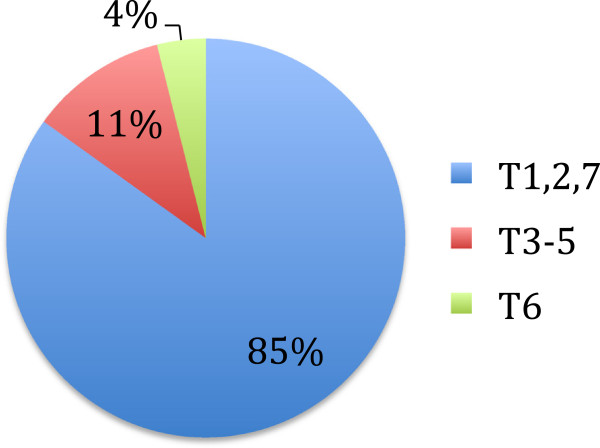
Medical students’ combined critical thinking response types: 85% were simple responses, 11% advanced thinking responses and 4% topic related spontaneous questions.

‘Simple responses’ , having no comments about the syllabus topics, comprised 85% of the response types, ‘advanced thinking responses’ contained 11%, and topic-related spontaneous questions were 4% for a total of 15% more advanced types of critical thinking directly related to the syllabus topics (Figure 1).

**Figure 2:**
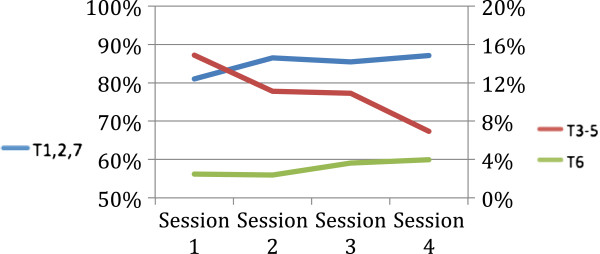
Type and frequency of medical students’ comments across the 4 sessions suggesting their comments decreased over time.

Examining the distribution of combined comments type for each session suggested that students’ comments generally decreased as time passed, giving little indication that critical analysis increased across time (Figure 2).

**Figure 3:**
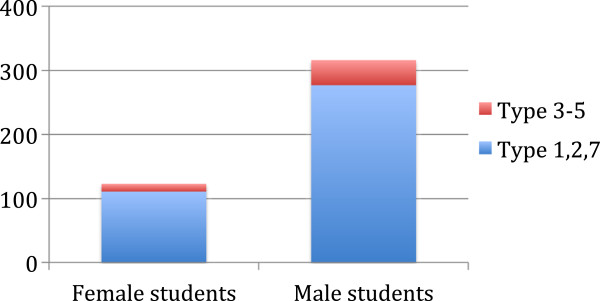
Distribution and number of comments by males and females were not statistically different.

In another analysis we combined types 1, 2 and 7 as ‘expanded simple responses’ and types 3 to 5 as ‘integration thinking’. Total comments made by students’ sex were proportionally represented with female students (n = 4) accounting for 33.0% (n = 142) and male students (n = 8) accounting for 67.0% (n = 278) (Figure 3). There was no statistical difference between male and female students (p = 0.69).

Students instructed by the female instructor also commented significantly more than those under the tutorage of a male instructor, with the female instructor's group contributing 60.0% of comments (n = 275).

**Figure 4:**
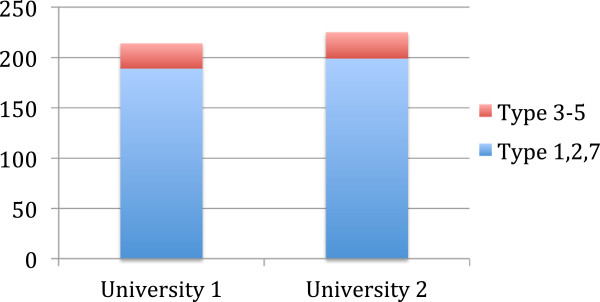
Distribution and number of comments by the universities were not statistically different.

We observed minor differences between the two medical universities, with University 2 students contributing more responses, regardless of their instructors (Figure 4). There was no statistical difference between students of University 1 and those of University 2 (p = 0.92).

## Discussion

We presented the first CA of medical students’ webinars. This is a unique application of the CA technique that was developed for health educators, to medical student seminars. The most basic level of simple responses having no comments about the syllabus topics, accounted for a majority (85%) of the total students’ comments. Only 15% of the students’ comments represented more advanced types of critical thinking.

It was anticipated that data would show improved clinical thinking as measured by Types 3 to 5 changes. Such was not the case. In part the total talking time of the students was very low, much lower than anticipated, with no change over the four sessions. Conversely the dominant talking time of the instructors implied they reverted to the traditional practice of lecturing, perhaps because they lacked skills to promote discussion.

Usual communication skills training and assessment of medical students center on interactions and feedback from standardized actor patients- objective structured clinical examination (OSCE) [17,18]. A two-day instructional communication skills report showed 5^th^ grade Japanese medical students’ marginal improvement using OSCE [19].

However, in medical educational communities where prior educational exposure has attenuated student responses, the seminar experiences requiring verbal interactions may be necessary before adequate doctor-patient communication skills develop.

Limiting factors of this study were small sample size, short interval for discussions and small number of sessions (four). We had envisioned a larger medical student sample size. However sample size was limited by the coordination problems expressed by several Japanese medical universities to participate. The duration of the study and small number of sessions were restricted by the tutors’ already too busy daily workloads.

There are many possible reasons for the short times of students’ advanced thinking responses. For the instructors their possible reasons were: 1) the instructors were not trained to teach PBL. PBL faculty development with practice including instant feedback, is not offered in Japanese medical universities; 2) Completion of a US residency does not provide adequate PBL coaching for medical students, a major criteria for instructor selection in our study; 3) The authors provided no inter-session improvement feedback; 4) The instructors likely felt compelled to complete the seminars’ syllabus, thinking it was more important than student participation; and 5) If the total number of tutored sessions had been increased to eight sessions over eight weeks, it is possible more critical thinking responses would have been observed.

For the students their possible reasons for their short times of advanced thinking responses were: 1) The students lacked prior long term educational experiences in verbal problem solving exercises; 2) Their usual ways of classroom learning has been limited to large hall lectures, a usual cultural norm; 3) The presence of a faculty member has been an inhibiting influence since silence shows respect to one’s elders; 4) An Asian student does not verbally challenge the instructor; 5) Speaking out has been considered culturally rude [11,12]; 6) The lack of familiarity with the tutors and relatively limited four sessions likely inhibited the students’ responses as suggested in a recent report [20].

Although the tutoring sessions did not appear to increase expression of PBL understanding over time, the students’ sex may have play a role in critical thinking. Within the scope of our very limited data, although the male students responded more, female students expressed proportionally more upper-level critical thinking than their male counterparts; the female instructor’s group also contributed significantly more comments, suggesting that Japanese women, who traditionally have been observed to be more expressive and nurturing may find it easier to grasp PBL concepts. However, these data were limited to verbal expressions; it may not be a fair representation of true levels of cognitive activity.

Also, that University 2 students generally commented more than University 1 students, suggested local medical education styles may influence students’ respond in web-tutoring sessions. Specifically University 2 actively promotes PBL by having faculty use their PBL teaching methods acquired during PBL workshops at an American medical school, and by encouraging students to participate in their popular extra-curricular PBL club. University 1 does not offer PBL faculty development or PBL clubs. However several clinical departments at University 1 offer PBL training to their postgraduate trainees.

Nonetheless, the application of CA, from the field of health and nutrition education, has the potential to make important contributions to improving clinical thinking skills, in both Western and non-Western medical universities.

Furthermore, detailed evaluation of faculty and student performance during small group seminars, CA may be ideally suited for this task given its many year multidisciplinary history [20-23]. Our report builds on the large number of CA studies in the scientific and educational literature. For example the nursing and nutritional health literatures are enriched by many CA studies [24-27]. Recent applications of CA include marketing drugs to women [28], and designing nutritional educational materials [29]. In the current milieu of interdisciplinary education, the inclusion of health and nutritional students and faculty, the groups actively using CA, have not been highlighted [30,31]. Also recent medical educator physician critics of PBL [32] and PBL defenders [33] may find benefit from applying CA to PBL seminars.

Based upon this analysis, future studies with more adequate instruction, larger sample sizes, and with longer duration may be able to demonstrat**e** that CA be a universal instrument to study the impact and improvements of PBL on training medical students in clinical thinking.

## Conclusions

In conclusion CA was applied to a multi-university real time PBL medical student clinical training webinar. Although the results are preliminary, mostly limited by the small sample size and short duration of the study, the CA technology may have the potential to improve PBL training for medical students; the challenges presented here warrant further investigation.

### Ethical approval

Ethical approval for educational studies and surveys is not required according to national practice in Japan. However, the study adhered to ethical principles, and the respondents are not identifiable from the data.

## Abbreviations

PBL: Problem-based learning; CA: Content analysis; Webinar: Web-based seminar; PIM: Practical inquiry model; OSCE: Objective structured clinical examination.

## Competing interests

The authors declare that they have no competing interests.

## Authors’ contributions

YT conceived of the study and designed the coding tables; GHS, KE and YTakata drafted the manuscript, KE, AA and YTokuda collected, coded and analyzed the data; SK, YK, HH, HK, HTokunaga MO, TM, HK and HTerasawa participated in the design and coordination of the medical students and technical recordings. All authors read and approved the final manuscript.

## Authors’ information

Yukari Takata is a senior evaluation specialist at IQ Solutions in Rockville, Maryland. She recently received the PhD in health education and mass communications at the College of Journalism and Communications, University of Florida, Gainesville, Florida, USA.

Gerald H. Stein is a Clinical Assistant Professor at the Department of Medicine, School of Medicine, University of Florida, Gainesville, Florida, and a Physician Consultant at the Clinic of Jurisdiction, Veteran Affairs Medical Center, Gainesville, Florida, USA.

Kuniyuki Endo is a staff physician at the Department of Neurology, Nagoya Daini Red Cross Hospital, Nagoya, Aichi, Japan.

Akiko Arai is a candidate for the PhD at the College of Health and Human performance, University of Florida, Gainesville, Florida, USA.

Shun Kohsaka is an Assistant Professor at the Department of Cardiology, School of Medicine, Keio University, Tokyo, Japan.

Yuka Kitano is an Assistant Professor at the Department of, Emergency Medicine and Critical Care Medicine, School of Medicine, St. Marianna University, Kawasaki, Kanagawa, Japan.

Hitoshi Honda is an Attending Physician and consultant, Departments of General Internal Medicine and Infectious Diseases, Teine Keijinkai Medical Center, Sapporo, Hokkaido, Japan.

Hidetaka Kitazono is an Attending Physician and consultant, Departments of General Internal Medicine and Infectious Diseases Tokyo Bay Medical Center, Noguchi Hideyo Memorial Noguchi International Hospital, Urayasu, Chiba, Japan.

Hironobu Tokunaga MD, is an Assistant Professor at the Department of Emergency Medicine, University of Fukui Faculty of Medical Sciences, Fukui, Japan.

Yasuharu Tokuda, MD, MPH, is a Professor at the Institute of Clinical Medicine, Graduate School of Comprehensive Human Sciences, University of Tsukub, School of Medicine, Tsukuba, Japan.

Mikako Obika, MD, is an Assistant Professor at the Center for Graduate Medical Education, Okayama, Okayama University Medical School, Japan.

Tomoko Miyoshi, MD, is a Professor at the Department of Medical Education and Primary Care, Okayama University Medical School, Okayama, Japan.

Hitomi Kataoka, MD, is a Professor at the Department of Medical Education and Primary Care, Okayama University Medical School, Okayama, Japan.

Hidekazu Terasawa, MD, is a Professor at the Department of Emergency Medicine, University of Fukui Faculty of Medical Sciences, Fukui, Japan.

## Pre-publication history

The pre-publication history for this paper can be accessed here:

http://www.biomedcentral.com/1472-6920/13/156/prepub
